# Hospital Administration

**Published:** 1896-10-03

**Authors:** 


### HOSPITAL ADMINISTRATION.

### STATE SUPPORT AND POLITICS.

### THE ADELAIDE HOSPITAL AND THE GOVERNMENT OF SOUTH AUSTRALIA.

Among the remedies which have been prescribed for the chronic difficulties of the metropolitan hospitals the suggestion that they should be converted into State-supported institutions is one of the most specious. It is easy to understand that the members of boards of management, constantly hampered and harassed by want of money, may in faint-hearted moments be ready to welcome a scheme which would cut the Gordian knot; but it is difficult to conceive how any member of the medical profession should be able to convince himself that a State Department, fed by the rates, would be a better or a more generous form of maintenance than the free gifts of a benevolent public. The spectre of "Hospital Abuse" has, however, so got upon the nerves of some that they are ready to resort to any incantation. They are so blind to the lesson taught by the course of conduct and standard of action commonly displayed by boards of guardians and the sanitary authorities towards their medical and nursing officers that they are ready to surrender the whole future of medicine and medical education into the hands of an organisation where two leading principles would be economy and political expediency.

The history of the present controversy with regard to the Adelaide General Hospital, South Australia, affords so striking an illustration of some of the evils which must flow from a system which makes hospital management one of the planks in a political platform that it is worth while to relate the circumstances at some length.

All the hospitals in the Colony of South Australia, with the exception of the Adelaide Children's Hospital, are supported almost entirely by the State.* They are controlled by the Chief Secretary, who is the responsible head of the whole system. An honorary board of

* For the facts of the controversy reliance is placed chiefly on a pamplet issued by the South Australian Branch of the British Medical Association. It is entitled, "The Adelaide Hospital, Adelaide, South Australia. A short Account of the Circumstances which led to the Resignation of the Honorary Staff." Adelaide: 1896.

management is appointed to regulate the internal working of each hospital. For close on thirty years the Adelaide Hospital has been managed by a board of sixteen members, which, at the time the recent trouble arose (in 1894), comprised, Mr. Graves (chairman), who had served on the board for over twenty years; Sir John Colton, a veteran statesman and philanthropist; three well known and greatly respected M.P.'s; an ex-M.P.; the Mayor and one of the Aldermen of Adelaide; three medical practitioners not on the honorary staff of the hospital; and three senior members of that staff. Everything worked very smoothly; the recommendations of this responsible board invariably received the Government sanction, which had, in fact, come to be looked upon as a mere formality. The board had raised the number of beds in the hospital to 300, and had introduced many reforms required by modern views as to hospital management, so that, at the date when political considerations induced the Government to upset this system of management by a voluntary committee, Adelaide possessed a hospital which compared very favourably with any hospital in any part of the world, an efficient and painstaking medical superintendent, a nursing staff admirably organised and well disciplined, and a distinguished honorary medical and surgical staff, the senior physician being Dr. J. C. Verco, and the senior surgeon, Dr. G. C. Stirling, C.M.G., F.R.S. For eleven years the hospital had served for the clinical teaching of the Medical Department of the University of Adelaide, the number of students being about fifty.

In September, 1894, in a sudden spasm of economy, the Government cut down the salary of the superintendent of nurses from £120 to £100. The office was vacant at the time, and Miss McLeod, superintendent of night nurses, was appointed to it. The Chief Secretary then gave orders to the board that the office of superintendent of night nurses must be filled from among the nurses in the Government service. The board, on the advice of the medical superintendent, and with the concurrence of the superintendent of nurses, chose Miss Gordon, a junior charge nurse. This appointment was resented by some of the charge nurses, who made an energetic protest at the next meeting of the board. The board made a courteous reply, adhering to its opinion that Miss Gordon's appointment would advance the best interests of the hospital.

Anything like collective action or protest by the nurses ceased at this point, the claim of the board to select the nurse believed to be most competent being too obviously well founded. One nurse, however, a few weeks senior to Miss Gordon, was very indignant, and was joined in her protests by a probationer nurse, who seems to have had no semblance of a grievance. Their cause was taken up by a section of the press, which published leading articles condemning the board in the strongest terms, and "opened its columns liberally to a perfect deluge of correspondence attacking Dr. Perks (the medical superintendent) and the management with a degree of bitterness impossible to surpass." A special animus was given to the discussion by the fact, of which the majority of the board were unaware when the appointment was made, that Miss Gordon was a sister of the Chief Secretary. The Premier now took the matter in hand and endeavoured to convince the board that a seniority of a few weeks was conclusive proof of the superiority of the one nurse over the other for the appointment in question. Finally, after the exchange of a series of memoranda and minutes, the appointment of Miss Gordon was confirmed by the Government, after an enquiry had been held by the board. At this enquiry the probationer (Miss Graham) behaved in such a manner that the board, deeming that her conduct before their committee was unsuitable, cancelled their recommendation that she should be promoted to be a charge-nurse. Her term of office as probationer had expired, and as she was not recommended for promotion she had no status, and ceased to be eligible for employment. As to the nurse (Miss Hawkins), who thought she ought to have been promoted to be night superintendent, she persisted before the committee in her charges against the medical superintendent that he was "untruthful, mean, unfair, and hypocritical"—statements which the committee reported they "confidently believe to be incorrect." The board therefore recommended the dismissal of Nurse Hawkins.

The Government, yielding to the clamour of one section of the press, refused to conform to the findings of the board, and appointed a Royal Commission "to inquire into the management and condition of the Adelaide Hospital." The controversy had now become a burning political question, and the Commission consisted of the Treasurer of the Colony, a lawyer who had already advocated the nurses' cause, two M.P.'s, who represented the Trades and Labour Council, a body which, we are told, had already passed a resolution tantamount to condemnation of the board—a Government surgeon, a lady, and a Government supporter in the Upper House. The conduct of the inquiry and the impartiality of the Commission was open to criticism, but in their final report they found "that generally the institution is admirably conducted. No requirement essential to the well-being of the patients has been overlooked, and very much excellent work has been and is being accomplished at reasonable cost." Nevertheless, the Commissioners in an interim report had been willing to jeopardise the discipline and, therefore, the efficiency of this institution, so admirably conducted, and so successful in providing for the well-being of its patients, by recommending that both Nurse Hawkins and Probationer Graham should be re-employed. That their conduct had been without excuse is shown by the fact that even this friendly commission found it necessary to make their reinstatement conditional "on the withdrawal of the offensive expressions against the Government, the hospital board, and its officers."

About this time the South Australian branch of the British Medical Association held a meeting which was largely attended. At this meeting a resolution was adopted expressing entire approval of the conduct of the board of management and strong sympathy with the medical superintendent "in the gross and unfounded charges made against him in the evidence of certain witnesses before the Royal Commission."

The board protested against the decision of the Government, that the insubordinate nurse and probationer should be appointed to the hospital, and pointed out that under the Act of Parliament the general management and superintendence of the hospital rested with it and not with the Government. The Premier remained obdurate, and the board finally appointed Nurse Hawkins to have charge of a ward, but refused to appoint Probationer Graham a charge nurse. The reason for this partial surrender was that the board considered that they had no legal power to refuse to take back Nurse Hawkins, but that the probationer had no legal status, and that in her case, therefore, they could follow their considered opinion.

The Government, however, would have no compromise, and on August 29th, 1895, informed the board that they would not be reappointed when their term of office expired in March, 1896. Probationer Graham was given six months' leave of absence on full pay. On September 5th the medical superintendent resigned. In February, 1896, the superintendent of nurses resigned. She found it difficult to maintain proper order and discipline, which is not surprising, seeing that the Premier and the Cabinet had usurped her functions. The immediate cause assigned was the conduct of a probationer who was impertinent, and also addressed an insulting letter to the Premier. In the course of this letter the probationer implored the Premier to protect her against the board of management, "of whom no nurse can expect either justice or mercy." There was a good deal more in the same style, but as this probationer subsequently confessed that the letter was written for her, apologised, and left the hospital, it is unnecessary to quote from it further. Before this the night superintendent of nurses (Miss Gordon) had resigned, also for reasons similar to those which actuated Miss McLeod.

We have now reached the final stage of this instructive but rather tedious history. The climax occurred when on the last day of February the term of office of the old board expired. During the first week of March, 1896, when the new board had not yet come into office, the Government, donning the cap and frock of a superintendent of nurses, appointed Probationer Graham to be a charge nurse, and placed her in charge of a ward. The new board, when its constitution was announced, was found to contain the name of a medical man to whose conduct serious exception had been taken by the profession in the colony, and who had in consequence ceased to be a member of the South Australian branch of the British Medical Association. This gentleman, finding that his presence was distasteful to other members, did not long retain his seat on the board. The medical staff, however, felt that their position had become untenable; the Government claimed the right to appoint direct to offices in the hospital, and had refused to the medical staff any representation on the board of management. That the action of the staff was approved by the medical profession in the colony was shown by the fact that when the vacant appointments were advertised a totally inadequate number of applications was received. The Premier, in an election speech, had referred to the gynæcologist to the hospital as a "medical Jack the Ripper," and had made very disparaging remarks as to the profession as a whole. Endless side issues had been introduced; the attack on the board was clearly animated by political considerations, and not by any anxiety for the welfare of the patients or any acquaintance with hospital requirements.

As a result we find that this large hospital is now completely under the control of a Government which depends greatly on the support of the Labour party. Insubordinate nurses have, clearly for political purposes, received the sympathy and support of this powerful political organisation. Two women occupying subordinate positions have been enabled to insult the board of management and their nursing superiors, and have been rewarded for their contumacious conduct. Those who during many years had raised the standard of efficiency of the hospital, and had maintained it at a high level, have been treated most ungratefully. Finally, the medical faculty of the university has been deprived of its clinical hospital, and its future is thus endangered.

All this has come about not in the interest of the patients of the hospital, nor of the nurses, nor of the medical staff, but solely because the Labour party wanted a stick with which to beat the Government, and the Government has been afraid to stand its ground.

A resident surgeon and a resident physician, at salaries of £400 and £300 a year respectively, have been obtained from England, and they are now in charge of this hospital of 300 beds. Whether the Premier is called into consultation before an operation is performed, and whether the physician's prescriptions are revised at meetings of the Cabinet Council, history sayeth not. But it cannot be thought that the condition of the Adelaide Hospital is as satisfactory as it was two years ago, when it had an experienced board of management, a zealous and efficient staff of seventeen medical officers, and a loyal and disciplined nursing staff.* As an illustration of the way in which the State support of hospitals may become mixed up with politics, to the grave disadvantage of the hospitals, the incident may serve a useful purpose as an awful example.

* Since this article was written it has been announced, by cable, from Adelaide, that all the House Surgeons to the hospital have resigned as a protest against the way in which the clinical work is now conducted.

